# Stabilization of symptomatic carotid atherosclerotic plaques by statins: a clinico-pathological analysis

**DOI:** 10.1007/s00380-018-1193-6

**Published:** 2018-05-22

**Authors:** Takao Konishi, Naohiro Funayama, Tadashi Yamamoto, Daisuke Hotta, Ryota Nomura, Yusuke Nakagaki, Takeo Murahashi, Kenji Kamiyama, Tetsuyuki Yoshimoto, Takeshi Aoki, Shinya Tanaka

**Affiliations:** 1 Department of Cardiology, Hokkaido Cardiovascular Hospital, 1-30, West 13, South 27, Chuou-ku, Sapporo, 064-8622 Japan; 2 Department of Cancer Pathology, Hokkaido University Graduate School of Medicine, Sapporo, Japan; 3 Department of Neurosurgery, Nakamura Memorial Hospital, Sapporo, Japan; 4 Department of Neurosurgery, Kashiwaba Neurosurgical Hospital, Sapporo, Japan; 5 Department of Neurosurgery, Hokkaido Neurosurgical Memorial Hospital, Sapporo, Japan

**Keywords:** Statin, Carotid artery disease, Carotid endarterectomy, Carotid plaque, Unstable plaque

## Abstract

Human and animal studies have revealed a stabilization of atherosclerotic plaques by statins. However, the stabilization of human carotid plaques has not been thoroughly described pathologically. This analysis explored the relationship between statin therapy and plaque stability in carotid endarterectomy (CEA) specimens. We analyzed specimens harvested between May 2015 and February 2017, from 79 consecutive patients presenting with > 70% carotid artery stenoses, of whom 66 were untreated (group 1) and 13 treated (group 2) with a statin. Immunohistochemistry was performed, using an endothelial specific antibody to CD31, CD34 and platelet derived growth factor receptor-β. The prevalence of plaque ruptures (*P* = 0.009), lumen thrombi (*P* = 0.009), inflammatory cells (*P* = 0.008), intraplaque hemorrhages (*P* = 0.030) and intraplaque microvessels (*P* < 0.001) was significantly lower in group 2 than in group 1. Among 66 patients presenting with strokes and infarct sizes > 1.0 cm^3^ on magnetic resonance imaging, the mean infarct volume was significantly smaller (*P* = 0.031) in group 2 (4.2 ± 2.5 cm^3^) than in group 1 (8.2 ± 7.1 cm^3^). The difference in mean concentration of low-density lipoprotein cholesterol between group 1 (121 ± 32 mg/dl) and group 2 (105 ± 37 mg/dl) was non-significant (*P* = 0.118). This analysis of plaques harvested from patients undergoing CEA suggests that statin therapy mitigates the plaque instability, which, in patients presenting with strokes, might decrease infarct volume.

**Electronic supplementary material:**

The online version of this article (10.1007/s00380-018-1193-6) contains supplementary material, which is available to authorized users.

## Introduction

Statins slow the progression of coronary atherosclerosis and lower the incidence of ischemic strokes [1]. Moreover, some studies have suggested that they lower total and ischemic cerebrovascular mortality, in secondary and primary prevention [2, 3]. With the prescription of 80 mg of atorvastatin daily, Amarenco et al. observed a 16% reduction in the rate of fatal and non-fatal strokes and a 26% reduction in the rate of overall vascular events [2]. Therefore, the interest in the effects of statins on the morphology and functional characteristics of atherosclerotic carotid plaques in humans and animals, has been growing. In a pathological analysis of neointima and media of rabbit femoral arteries, an arterial infiltration by macrophages was abolished and the concentrations of monocyte chemoattractant protein-1 were significantly decreased by statin therapy [4]. In a mouse model, statins have decreased the retinal neovascularization [5]. However, pathological analyses of carotid atherosclerotic plaques in patients treated versus untreated with statins have not been directly compared. This study is a detailed pathological comparison of atherosclerotic lesions and plaque stability in patients who underwent carotid endarterectomy (CEA), previously treated versus untreated with a statin. In addition, in patients who had suffered a stroke, the infarct volume was measured by magnetic resonance (MR) imaging.

## Methods

### Sample population

We analyzed data collected in 71 men and 8 women > 30 years of age who, between May 2015 and February 2017, underwent CEA consecutively in the departments of neurosurgery of Nakamura Memorial Hospital, Kashiwaba Neurosurgical Hospital or Hokkaido Neurosurgical Memorial Hospital, in Japan. The indication for surgery was the presence of a > 70%, symptomatic carotid artery stenosis. Patients were classified as symptomatic if they had suffered an ischemic stroke or a transient ischemic attack within last 6 months. Carotid plaque modifications were observed within 3–12 months after the onset of statin therapy [6–10], 66 untreated patients were classified as group 1, and 13 patients treated with a statin for ≥ 6 months were classified as group 2. Two patients treated with a statin less than 6 months were excluded from this study. The mean age of group 1 was 73.8 ± 7.0 years and that of group 2 was 71.6 ± 5.4 years (ns). CEA specimens that had been severely damaged by their excision were excluded from this analysis.

This study, approved by the Ethics Committee of each participating medical institution, complies with the Declaration of Helsinki on ethical principles for medical research involving human subjects, and all patients granted their written, informed consent to participate.

### Histopathological examinations

CEA was performed, using standard surgical techniques with minimal handling of the specimens. The plaques were removed en bloc, fixed in 10% buffered formalin, transected transversely in 5-mm specimens, and embedded in paraffin. After the 3-μm transverse sections from each block were stained with hematoxylin–eosin and elastica-Masson (which stains elastin black and collagen and proteoglycans green), the most stenotic segment and the proximal and distal adjacent segments were examined. The sections were examined by two independent observers, one of whom was an experienced histopathologist unaware of the clinical status and identity of the patients. The sections were immunohistochemically stained with anti-CD31, anti-CD34 and platelet derived growth factor receptor-β antibodies, which recognize endothelial cells, to confirm the presence of microvessels in the plaque.

### Definitions of histopathological indices and semi-quantification of histopathological observations

Plaque rupture is an area of disrupted fibrous cap, where the overlying thrombus is in continuity with the underlying necrotic core [11]. A thrombus consists of laminated platelets or fibrin, with or without interspersed red and white blood cells. Lipid core is defined as amorphous material containing cholesterol crystals [12]. An intraplaque hemorrhage is microscopically visible blood and thrombus inside the plaque. An intraplaque microvessel is a lumen surrounded by a rim of endothelial cells, highlighted by immunostaining with anti-CD34 antibodies. We also confirmed the presence of endothelial cells by immunostaining with anti-CD31 and platelet derived growth factor receptor-β antibodies. Minimum fibrous cap thickness is the thinnest part of the fibrous cap among all the cross-sections of a plaque [12].

Using reproducible semi-quantitative scales, we classified and scored between 1 and 3 or 4, 10 microscopic, histological characteristics of unstable plaques (Table 1) [13–16]. The score of each plaque characteristics was calculated as the average of the three scores which were analyzed in the three consecutive sections in pathological images.

**Table 1:** Semi-quantitative classification and scoring of histologic characteristics of unstable plaques

Characteristics	Score			
	1	2	3	4
Plaque rupture	Plaque intact	Plaque probably intact or artefactual break in the fibrous cap due to surgical incision	Plaque probably ruptured; the site of rupture is not clearly visible, though a thrombus adherent to lipid is present in the lumen	Definitely ruptured plaque
Thrombus	Absent	Small	Large	
Lipid core	Absent	Small; < 25% of the overall plaque area	Large; ≥ 25% of the overall plaque area	
Fibrous tissue	Minimal	≤ 50% of all tissue	> 50% of all tissue	
Inflammatory cells	Absent	Occasional cell or 1 group of > 50 cells per section	2–5 groups of > 50 cells per section	>5 groups of > 50 cells or 1 group of > 500 cells per section
Foamy macrophages	Absent	< 50 cells per section	≥50 cells per section	
Intraplaque hemorrhage	Absent	Small	Large	
Calcification	Absent	Stippling only	Calcified nodules	
Intraplaque microvessels	Absent	< 10 per section	≥10 per section	
Overall instability	Definitely stable; predominantly fibrous plaque with thick, intact fibrous cap	Probably stable; single sign of instability, such as small hemorrhage or inflammation	Probably unstable; signs of instability present, such as inflammation, < 200 µm minimum fibrous cap thickness and large lipid core without rupture	Definitely unstable; presence of rupture, thrombus, large hemorrhage, inflammation and < 200 µm minimum fibrous cap thickness

### Magnetic resonance imaging

All patients presenting with strokes or transient ischemic attacks underwent T1, T2 and diffusion MR imaging, using a 1.5 T scanner. Images of 66 of the 79 symptomatic patients (55 in group 1 and 11 in group 2) were retrospectively reviewed and analyzed to identify the lesion location and infarct volume. Follow-up MR images were obtained 2–4 weeks after the onset of stroke. Supplemental Figure I shows the measurements of infarct volume by T2 images, as measured by computed tomography of intracerebral hemorrhage volumes [17], using the formula for an ellipsoid:

$$(4/3\pi \left( {a/2} \right)\left( {b/2} \right)\left( {c/2} \right),$$

where *a*, *b* and *c* are the respective diameters in three dimensions.

### Statistical analysis

Continuous variables are reported as means ± standard deviations (SD) and categorical variables as counts and percentages. The normality of distributions was assessed by the Kolmogorov–Smirnov test. Between-group differences were examined, using Pearson’s Chi square or Fisher’s exact test for categorical variables and Student’s *t* test or Mann–Whitney *U* test for continuous variables, as appropriate. A *P* value < 0.05 was considered to indicate statistical significance. The data were analyzed with the SPSS 22.0 statistical system software (IBM Corporation, Armonk, NY, USA).

## Results

### Clinical characteristics

The clinical characteristics of the two groups of patients are compared in Table 2. The prevalence of strokes was 83% in group 1 versus 85% in group 2 (*P* = 0.768). The mean concentration of low-density lipoprotein cholesterol was 121 ± 32 mg/dl in group 1 versus 105 ± 37 mg/dl in group 2 (*P* = 0.118). The other characteristics, including medications and concomitant diseases were likewise similar in both groups (Table 2). The doses and duration of the various statins administered in the 13 patients of group 2 are listed in Table 3.

**Table 2:** Clinical characteristics of group 1 (statin-untreated) and group 2 (statin-treated)

	Group 1 (*n* = 66)	Group 2 (*n* = 13)	*P*
Age (years)	73.8 ± 7.0	71.6 ± 5.4	0.213
Men	58 (88)	13 (100)	0.412
Diabetes mellitus	26 (39)	4 (31)	0.785
Hypertension	49 (74)	11 (85)	0.656
Dyslipidemia	53 (80)	13 (100)	0.180
Chronic kidney disease	17 (26)	3 (23)	0.884
Current smoker	19 (29)	7 (54)	0.151
History of:			
Transient ischemic attack or cerebral infarction	13 (20)	4 (31)	0.604
Coronary artery disease	9 (14)	3 (23)	0.657
Peripheral artery disease	3 (5)	1 (8)	0.636
Prior drug therapy			
Aspirin	5 (8)	3 (23)	0.234
Clopidogrel	4 (6)	1 (8)	0.825
Cilostazol	1 (2)	0 (0)	0.655
Days between stroke onset and carotid endarterectomy	50 ± 45	39 ± 33	0.384
Baseline laboratory results			
Glucose (mg/dl)	135 ± 50	137 ± 48	0.904
Cholesterol (mg/dl)			
Low-density lipoprotein	121 ± 32	105 ± 37	0.118
High-density lipoprotein	52 ± 12	51 ± 14	0.934
Low-density/high-density lipoprotein cholesterol	2.5 ± 0.9	2.2 ± 1.1	0.371
Triglycerides (mg/dl)	147 ± 74	187 ± 71	0.083

Values are mean ± SD or numbers (%) of observations

**Table 3:** Individual doses of various statins and duration of therapy

Patient number	Statin	mg/day	Duration of therapy
1	Pitavastatin	1.0	≥ 1 year
2	Rosuvastatin	2.5	≥ 1 year
3	Rosuvastatin	2.5	8 months
4	Pravastatin	10.0	≥ 1 year
5	Pitavastatin	1.0	≥ 1 year
6	Rosuvastatin	2.5	11 months
7	Rosuvastatin	2.5	≥ 1 year
8	Rosuvastatin	2.5	≥ 1 year
9	Rosuvastatin	2.5	≥ 1 year
10	Rosuvastatin	2.5	≥ 1 year
11	Rosuvastatin	10.0	≥ 1 year
12	Rosuvastatin	2.5	6 months
13	Atorvastatin	5.0	≥ 1 year

### Histopathological plaque characteristics

**Fig. 1:**
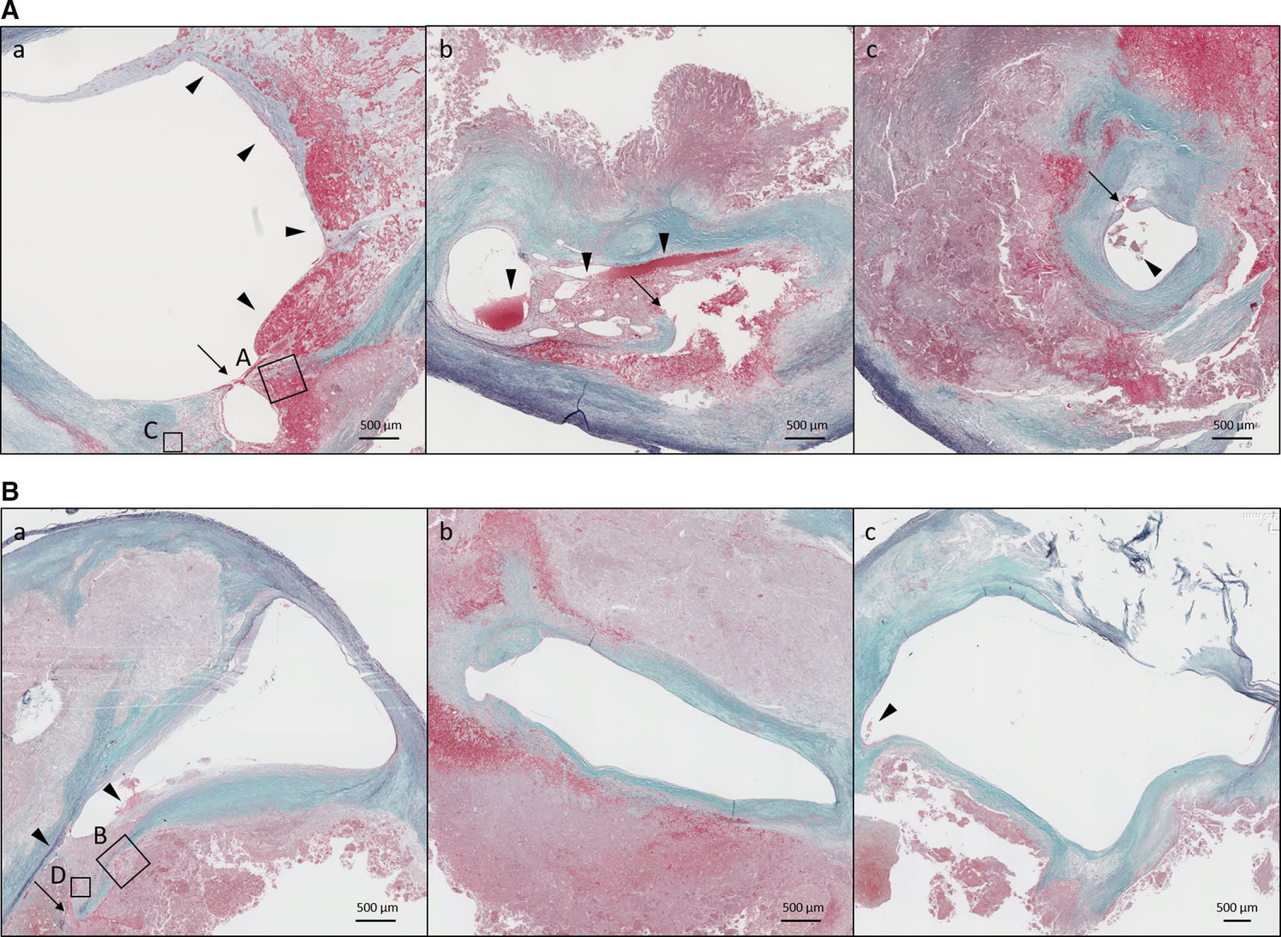
Representative microscopic plaque characteristics (low power images). **A** Ruptured plaque (**a**–**c** thin arrows) with large (**a**, **b** arrowheads) and small (**c** arrowhead) luminal thrombi harvested from a patient untreated with a statin. An extensive intraplaque hemorrhage is visible in each section. The squared area is magnified in Fig. 2a, c. Elastica–Masson staining. **B** Ruptured plaque (**a** thin arrow) with a large (**a** arrowheads) and small (**c** arrowhead) luminal thrombus stained in elastica-Masson from a patient treated with statins. A widespread (**b**) and comparatively focal (**a**, **c**) intraplaque hemorrhage is visible. The squared area is magnified in Fig. 2b, d. Eastica–Masson staining

**Fig. 2:**
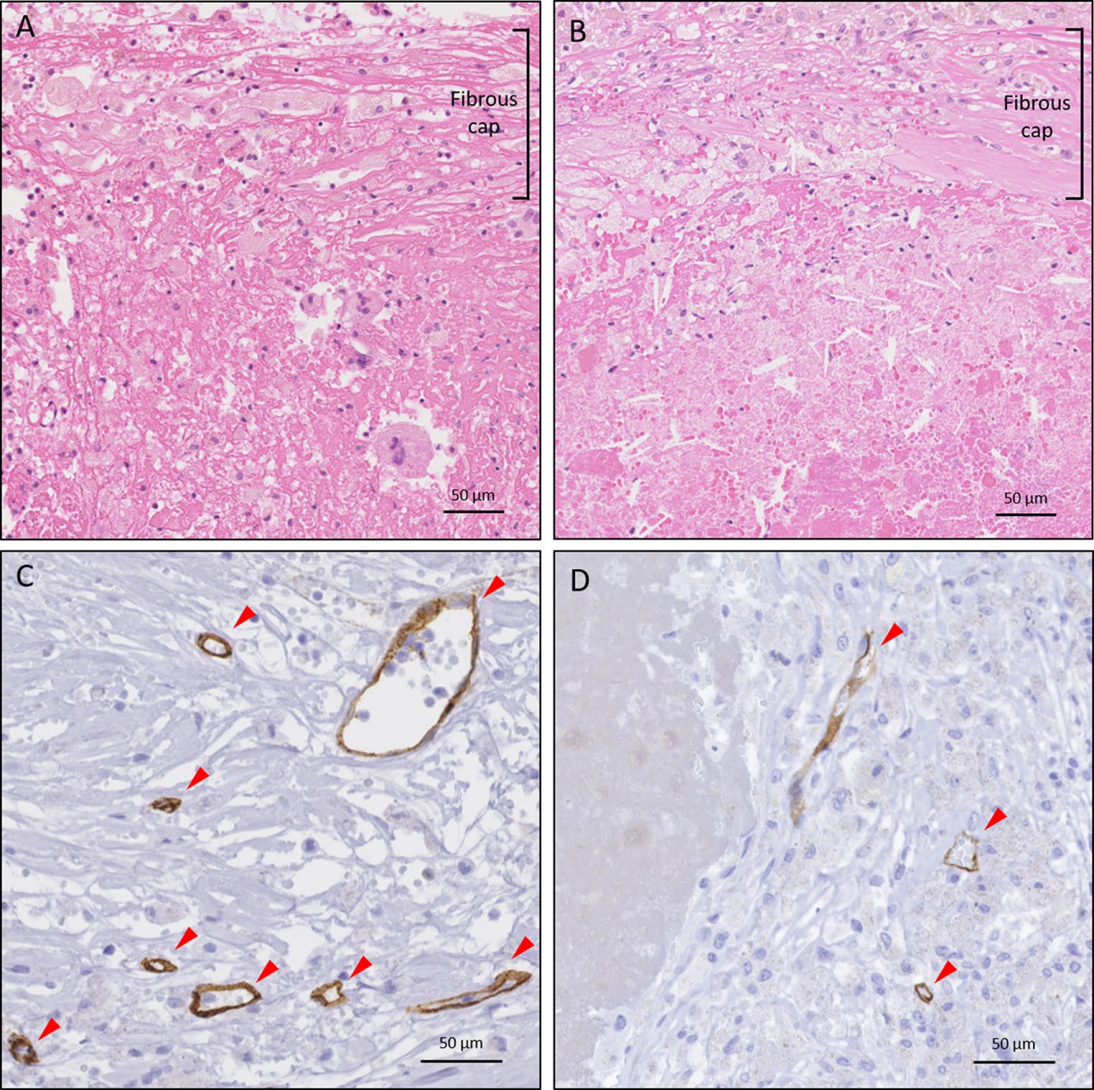
Representative microscopic plaque characteristics (high power images). **a**, **b** More prominent infiltration of inflammatory cells in **a** than in **b**. Hematoxylin–eosin staining. **c**, **d**. More prominent CD34 staining of intraplaque microvessels (red arrowheads) in **c** than in **d**

**Fig. 3:**
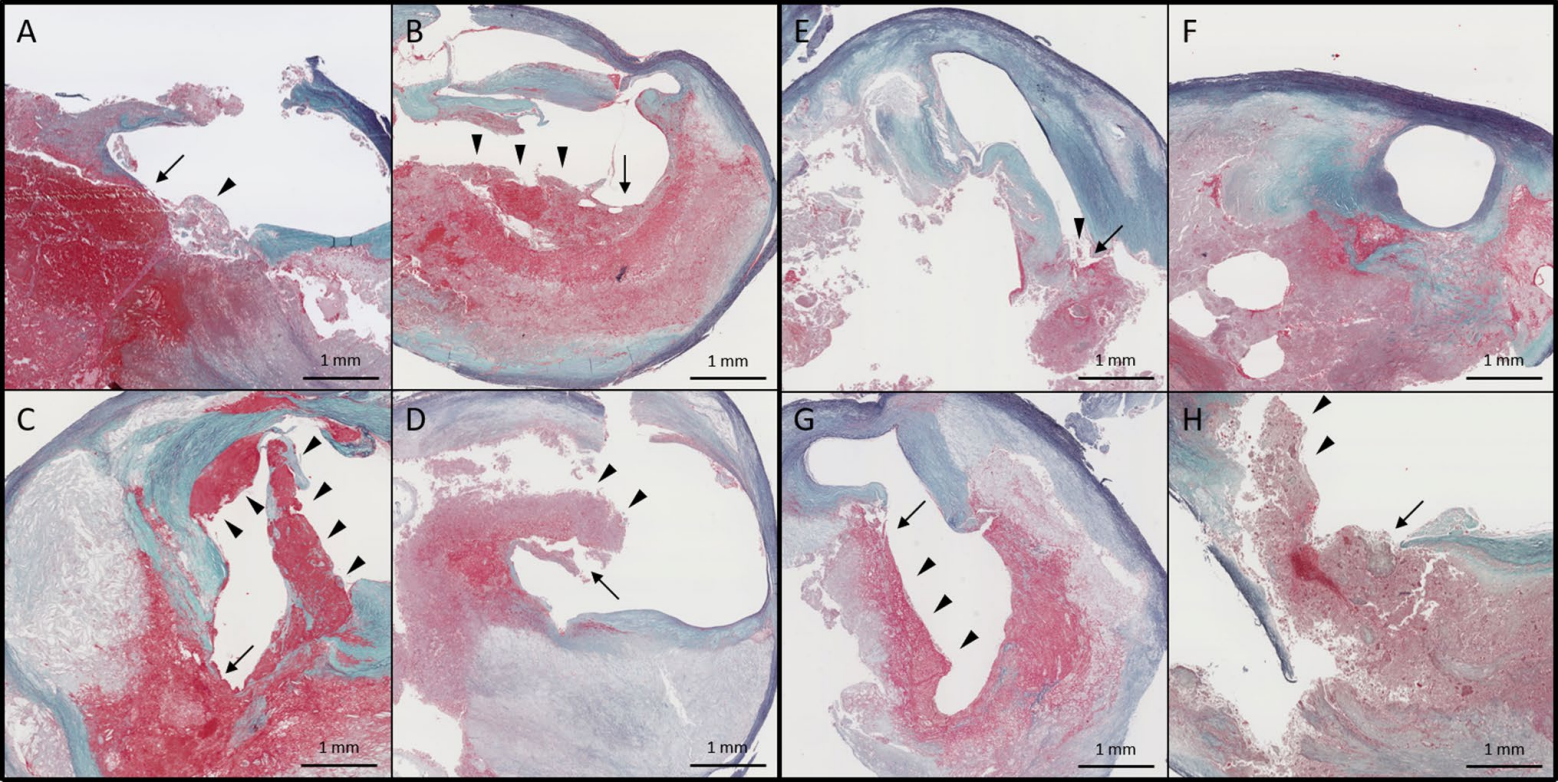
Representative microscopic plaque characteristics (low power images). Ruptured plaque (**a**–**e**, **g**, **h** thin arrows) with large (**b**–**d**, **g**, **h** arrowheads) and small (**a**, **e** arrowhead) luminal thrombi harvested from a patient untreated (**a**–**d**) or treated (**e**–**h**) with a statin. The cases of **a**–**d** in Fig. 3 correspond to those of **a**–**d** in Figs. 4 and 5, respectively

**Fig. 4:**
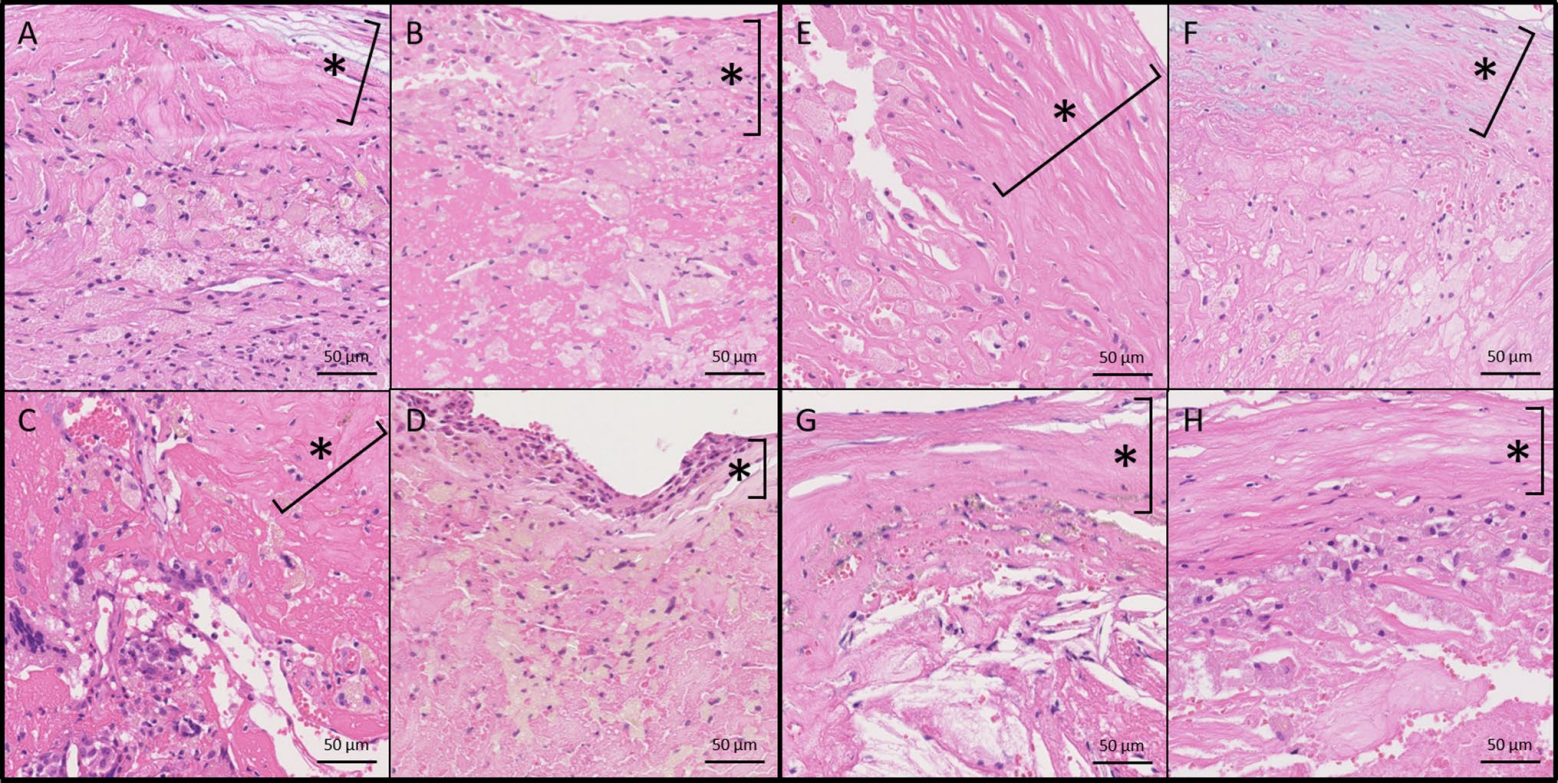
Representative microscopic plaque characteristics (high power images). More prominent infiltration of inflammatory cells in **a**–**d** than in **e**–**h**. Hematoxylin–eosin staining. Each asterisk indicates fibrous cap

**Fig. 5:**
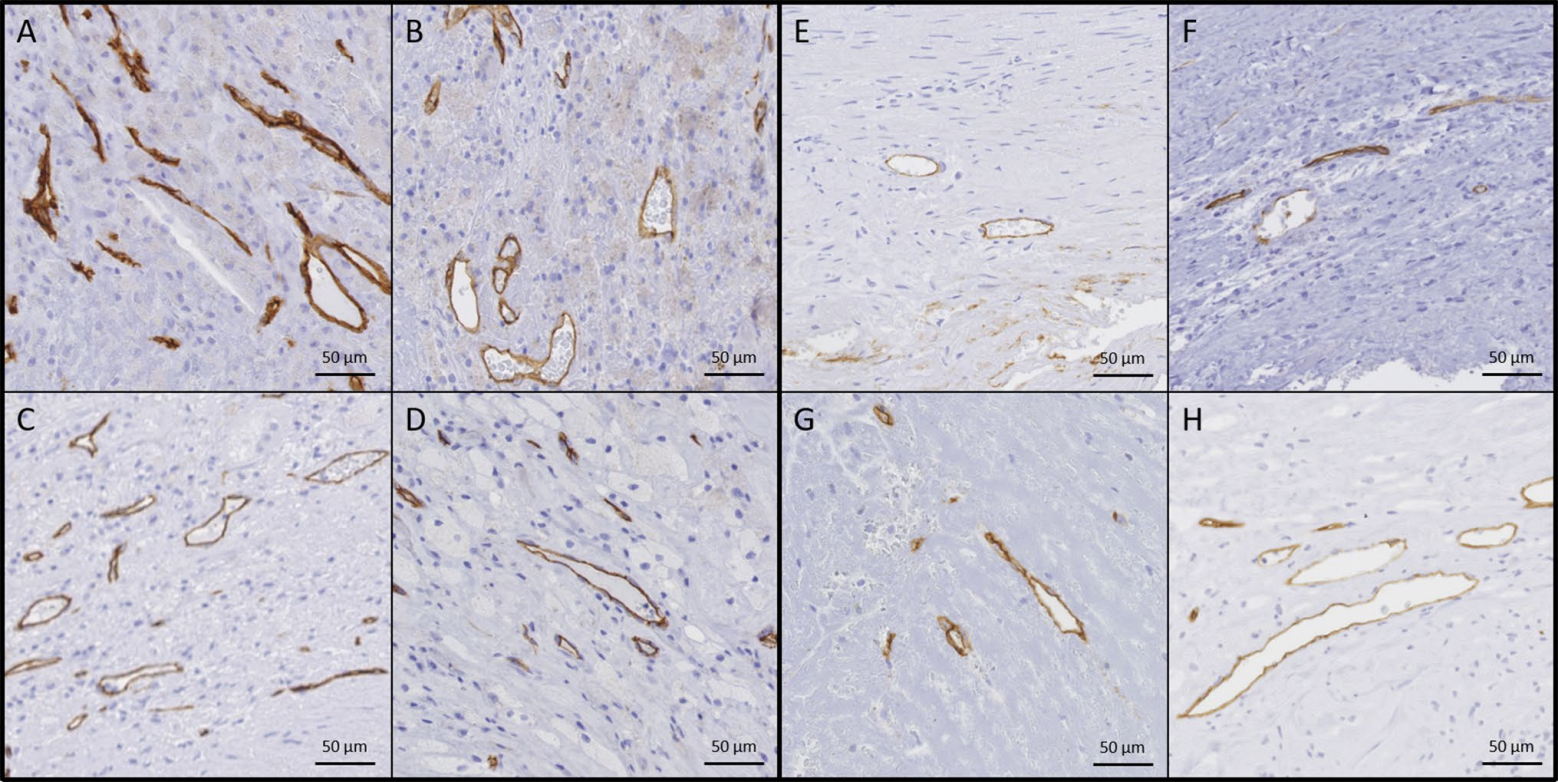
Representative microscopic plaque characteristics (high power images). More prominent CD34 staining of intraplaque microvessels in **a**–**d** than in **e**–**h**

The results of the semi-quantitative analysis of the various histopathological characteristics of the carotid plaques are compared in Table 4. Compared with group 1, the scores of plaque ruptures (*P* = 0.009), lumen thrombi (*P* = 0.009), inflammatory cells (*P* = 0.008), intraplaque hemorrhages (*P* = 0.030) and intraplaque microvessels (*P* < 0.001) were significantly lower in group 2. Furthermore, the mean number (26 ± 18 versus 51 ± 32 per section) and mean density (1.06 ± 0.84 versus 2.19 ± 1.43 per mm^2^) of intraplaque microvessels were significantly lower in group 2 than in group 1 (*P* < 0.001 for both comparisons). Representative examples of morphological differences between the two groups are shown in Figs. 1, 2, 3, 4 and 5.

**Table 4:** The scores of histological characteristics of group 1 (statin-untreated) and group 2 (statin-treated)

	Group 1 (*n* = 66)	Group 2 (*n* = 13)	*P*
Plaque rupture	2.82 ± 0.77	2.36 ± 0.48	0.009
Lumen thrombus	1.97 ± 0.42	1.69 ± 0.32	0.009
Lipid core	2.89 ± 0.24	2.79 ± 0.32	0.193
Fibrous tissue	2.38 ± 0.36	2.49 ± 0.35	0.319
Inflammatory cells	3.64 ± 0.48	3.31 ± 0.48	0.008
Foamy macrophages	2.84 ± 0.29	2.67 ± 0.41	0.088
Intraplaque hemorrhage	2.75 ± 0.39	2.49 ± 0.46	0.030
Calcifications	2.43 ± 0.62	2.72 ± 0.40	0.113
Intraplaque microvessels	2.88 ± 0.23	2.59 ± 0.34	< 0.001
Overall instability	3.29 ± 0.38	3.13 ± 0.26	0.098

Values are mean ± SD

**Fig. 6:**
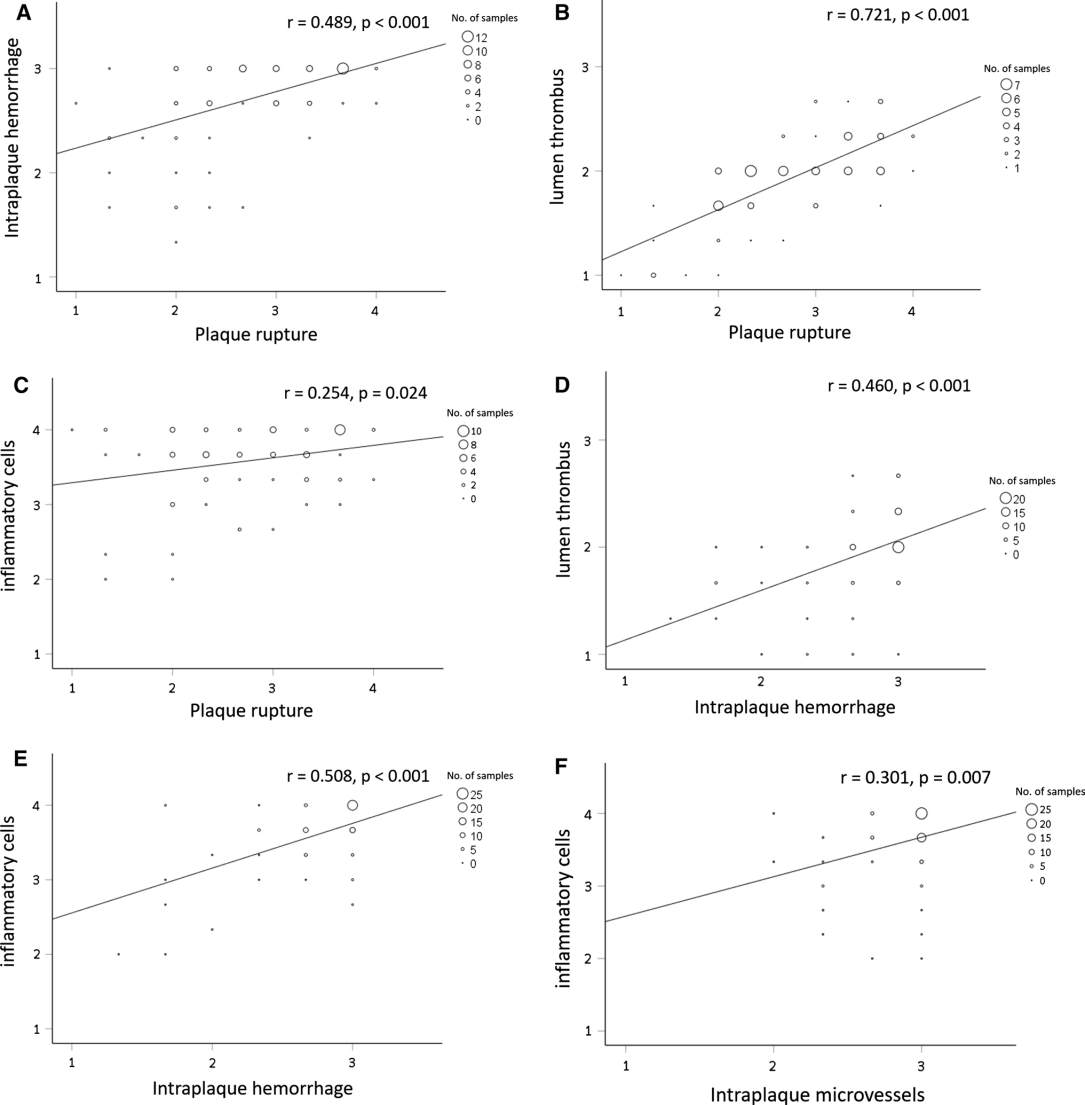
The relationships between scores of the plaque characteristics. Correlations between **a** plaque rupture and intraplaque hemorrhage (*r* = 0.489, *r*^2^ = 0.239, *P* < 0.001), **b** plaque rupture and lumen thrombus (*r* = 0.721, *r*^2^ = 0.520, *P* < 0.001), **c** plaque rupture and inflammatory cells (*r* = 0.254, *r*^2^ = 0.065, *P* = 0.024), **d** intraplaque hemorrhage and lumen thrombus (*r* = 0.460, *r*^2^ = 0.212, *P* < 0.001), **e** intraplaque hemorrhage and inflammatory cells (*r* = 0.508, *r*^2^ = 0.258, *P* < 0.001) and **f** intraplaque microvessels and inflammatory cells (*r* = 0.301, *r*^2^ = 0.090, *P* = 0.007)

Several correlations were observed among these plaque characteristics. Intraplaque hemorrhage (*r* = 0.489; *P* < 0.001), lumen thrombus (*r* = 0.721; *P* < 0.001) and inflammatory cells (*r* = 0.254, *P* = 0.024) were positively correlated with plaque rupture (Fig. 6a–c). Intraplaque hemorrhage was positively correlated with lumen thrombus (*r* = 0.460; *P* < 0.001; Fig. 6d) and inflammatory cells (*r* = 0.508; *P* < 0.001; Fig. 6e), and intraplaque microvessels was positively correlated with inflammatory cells (*r* = 0.301; *P* = 0.007; Fig. 6f). There was no correlation between intraplaque hemorrhage and intraplaque microvessels (*r* = 0.088; *P* = 0.442).

### Comparison of infarct volume in group 1 versus group 2

**Fig. 7:**
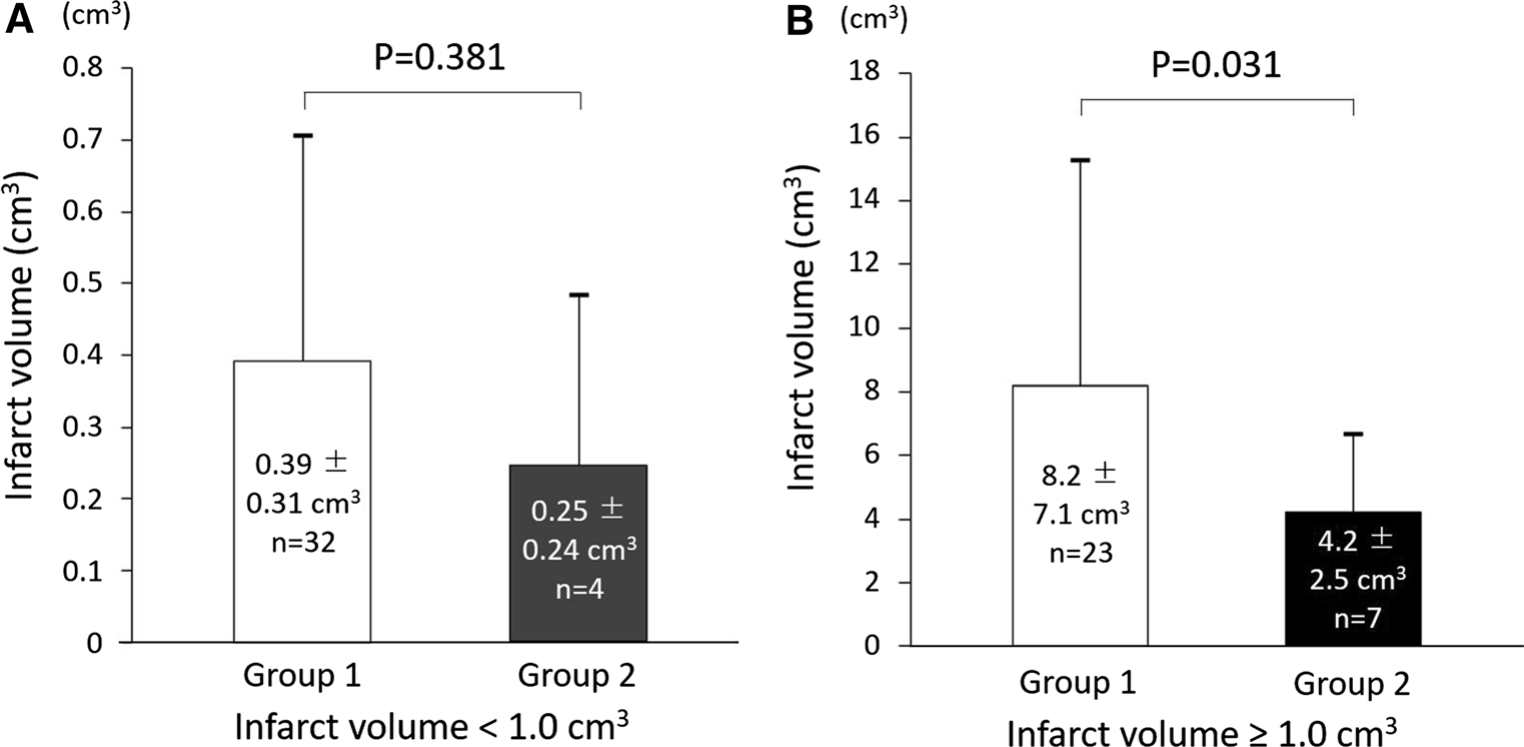
Comparison of infarct volume in patients presenting with strokes in group 1 versus group 2. Among patients presenting with strokes, whose infarct volume was ≥ 1.0 cm^3^, the mean infarct volume was significantly smaller (*P* = 0.031) in group 2 than in group 1

**Fig. 8:**
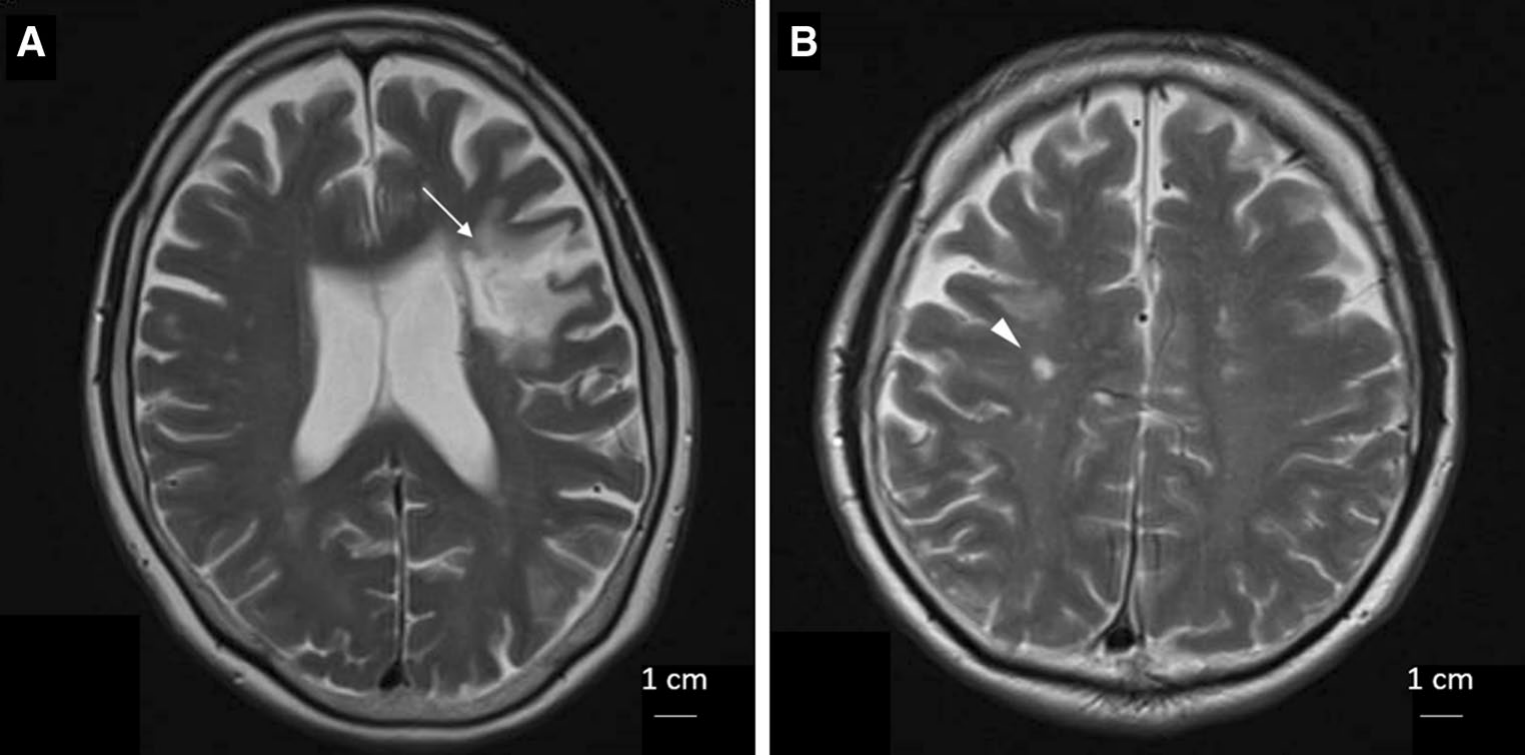
Representative MR images of the statin and no-statin patients. The infarct volume was measured as **a** 13.8 cm^3^ in a 60-year-old female without previous statin (arrow) and **b** 1.9 cm^3^ in a 69-year-old male with previous statin (arrowhead)

On follow-up MR imaging the mean infarct volume in the 66 patients who suffered strokes was 3.5 ± 5.5 cm^3^. In the subgroup whose infarct volume was < 1.0 cm^3^, the mean volume in group 2 (0.25 ± 0.24 cm^3^) was smaller than in group 1 (0.39 ± 0.31 cm^3^), though this difference was not statistically significant (*P* = 0.381; Fig. 7a). In contrast, in the subgroup whose infarct volume was ≥ 1.0 cm^3^, the mean volume in group 2 (4.2 ± 2.5 cm^3^) was significantly smaller than in group 1 (8.2 ± 7.1 cm^3^; *P* = 0.031; Fig. 7b). Representative MR images from both groups are shown in Fig. 8. The mean time interval between onset of stroke and follow-up MR imaging was similar in both groups (data not shown).

### Correlation between infarct volume and histopathologic plaque characteristics in patients presenting with strokes

**Fig. 9:**
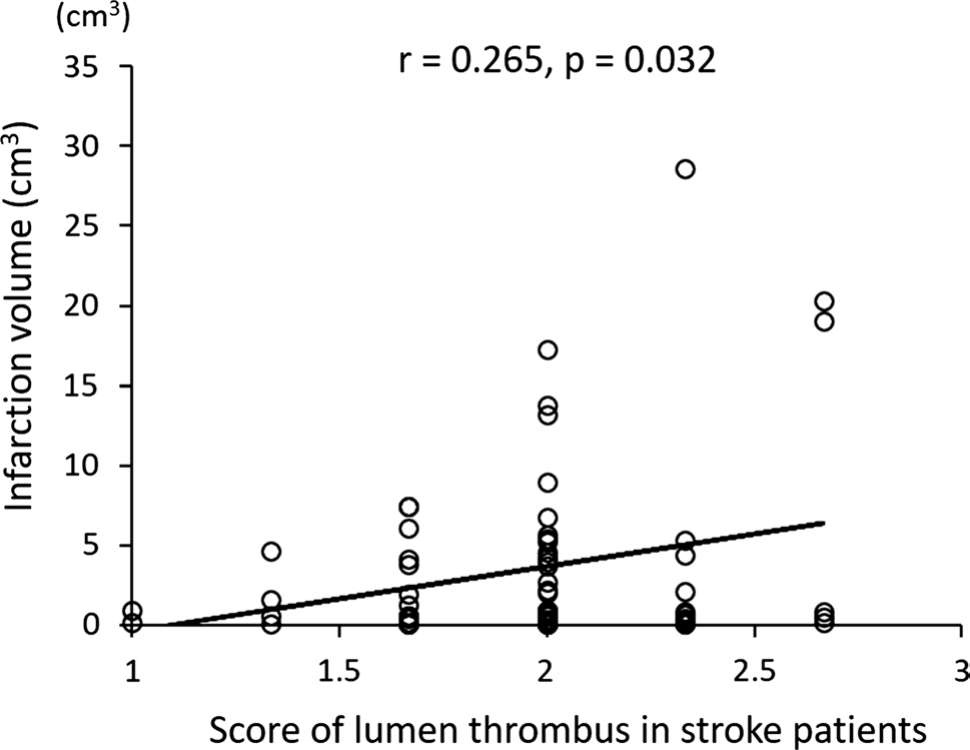
Correlation between infarct volume and semi-quantitative score of lumen thrombi in 55 patients in group 1 and 11 patients in group 2, presenting with strokes. In patients presenting with strokes, the infarct volume was positively correlated to lumen thrombus (*r* = 0.265; *r*^2^ = 0.070; *P* = 0.032)

In patients presenting with strokes, infarct volume was positively correlated with lumen thrombus (Table 5; Fig. 9). On the other hand, there was no correlation between infarct volume and the other histopathologic plaque characteristics (Table 5).

**Table 5:** Correlations between infarct volume and histopathologic plaque characteristics in patients presenting with strokes

	Infarct volume		
	*r*	*r* ^2^	*P*
Lumen thrombus	0.265	0.070	0.032
Plaque rupture	0.203	0.041	0.102
Lipid core	0.051	0.003	0.684
Fibrous tissue	− 0.055	0.003	0.663
Inflammatory cells	0.124	0.015	0.321
Foamy macrophage	0.119	0.014	0.340
Intraplaque hemorrhage	0.035	0.001	0.783
Calcification	− 0.011	< 0.001	0.929
Intraplaque microvessels	0.125	0.016	0.317
Overall instability	0.106	0.011	0.398

## Discussion

The main observations made in this study were: (1) in patients treated with statins, plaque ruptures, lumen thrombi, intra-plaque hemorrhages, intraplaque microvessels and inflammatory cells were significantly less prevalent than in patients untreated with statins, while the mean concentration of low-density lipoprotein cholesterol was similar in both groups; and (2) in patients presenting with strokes whose infarct size was > 1.0 cm^3^ on MR imaging, the mean infarct volume was significantly smaller in the group treated than in the group untreated with statins. To the best of our knowledge, this clinico-pathologic study is the first (a) in depth comparison of carotid artery plaques harvested from patients treated versus untreated with statins, and (b) analysis of the correlations among statin use, pathological characteristics and infarct volume in patients with strokes who underwent CEA. These observations further our understanding of the pathological characteristics of unstable plaques and of the pharmacological effects of statins on atherosclerotic plaques, which may have important implications with regard to the treatment of patients presenting with carotid artery stenoses.

### Statins, plaque rupture and intraplaque hemorrhage

The significantly lower plaque rupture score in patients who had received prior statin therapy than in those who had not been treated, suggests that the treatment narrowed the range of plaque ruptures. Endothelial dysfunction is a cause of plaque progression and a trigger of plaque rupture [18, 19]. Statins mitigate endothelial dysfunction in coronary and peripheral arteries of patients with or without dyslipidemia [20–22], and increase the concentrations and activity of endothelial nitric oxide (NO) synthase in human endothelial cells [23, 24]. They also improve vascular function by decreasing the production of endothelin [25]. Because inflammatory cells, such as macrophages and T cells, produce metalloproteinases and cytokines that weaken the tensile strength of the collagen cap, resulting in plaque rupture, less inflammation in atherosclerotic plaques might also attenuate the magnitude of plaque rupture (Fig. 6c). These effects might limit the size of plaque ruptures in patients treated with statins.

The lower prevalence of intraplaque hemorrhages in patients treated than in patients untreated with statins can be explained by fewer plaque ruptures. Figure 6a shows a positive correlation between intraplaque hemorrhages and plaque ruptures [26, 27]. Fewer plaque ruptures in the statin group might translate into fewer intraplaque hemorrhages from less luminal discontinuity, thus less penetration of blood into the plaque.

There are at least three explanations for the positive correlation between intraplaque hemorrhage and inflammation. First, intraplaque hemorrhages introduce neutrophils and mononuclear cells into the plaque, and leukocyte extravasate from the intraplaque microvessels [28]. Second, erythrocytes included in the intraplaque hemorrhages rapidly release large amounts of hemoglobin, which attract inflammatory cells to the atherosclerotic plaque [29]. Third, the migration of macrophages may be promoted by multi-specific receptors on erythrocyte membranes, including monocyte chemoattractant protein-1 [30]. Michel et al. indeed, observed a high density of macrophages involved in red blood cells and iron phagocytosis in intraplaque hemorrhages [28]. These observations explain the positive correlation we found between intraplaque hemorrhage and inflammatory cells.

While immature and leaky intraplaque microvessels are a known source of intraplaque hemorrhage [31], intraplaque hemorrhage in this study was probably due mostly to plaque rupture, since we found no correlation between intraplaque hemorrhage and intraplaque microvessels.

### Statin and lumen thrombus

Mean lumen thrombus was significantly smaller in patients treated with statins than in untreated patients. A decrease in the incidence of venous thromboembolisms associated with statin use has been reported [32–34]. Among patients undergoing CEA, statin therapy has been correlated with a reduced plaque expression of tissue factor, tissue factor pathway inhibitor antigens and tissue factor activity [35, 36]. Statins also increase the expression of the endothelial surface anticoagulant thrombomodulin by decreasing the activation of GTPase [37] and by increasing the concentrations of endothelium-derived NO [38]. Furthermore, thrombomodulin activates the protein C anticoagulant system, which inactivates bound factor Va [39]. These mechanisms and narrower range of plaque rupture (Fig. 6b) may decrease the size of thrombi in statin-treated patients. Since plaque rupture is a disruption of the fibrous cap with an overlying thrombus, and plaque rupture was positively correlated with intraplaque hemorrhage, one could expect a positive correlation between lumen thrombus and intraplaque hemorrhage.

### Statin and intraplaque microvessels

There are at least two mechanisms that may explain the decreased intraplaque microvessels in patients treated with statins. First, the treatment was associated with significantly lower plasma concentrations of vascular endothelial growth factor [40]. Second, since inflammation is one of the strongest stimuli of vessel growth [41], less inflammation might have contributed to the formation of fewer intraplaque microvessels in patients treated with statin. A positive correlation between intraplaque microvessels and inflammatory cells was, indeed, observed in this study.

Our observations of significantly fewer intraplaque microvessels in treated than in untreated patients are concordant with a previous report [42]. Among a sample of symptomatic and asymptomatic patients who had undergone CEA, Koutouzis et al. found significantly fewer intraplaque microvessels in patients who had been, than in patients who had not been treated with statins [42].

### Statins and inflammation

The inflammation score was significantly lower in patients treated with statins than in untreated patients. Atherosclerosis is a chronic inflammatory process of the vascular wall that is initiated by excessive LDL cholesterol and is mediated by inflammatory leukocytes such as activated macrophages, neutrophils, T lymphocytes and B lymphocytes [43]. Statin therapy is associated with lower cytokine levels in carotid plaques removed at the time of CEA [44]. Statins inhibit trans-endothelial migration of leukocytes by decreasing the expression of adhesion molecules such as intercellular adhesion molecule-1, lymphocyte function-associated antigen-1 and macrophage receptor-1, which all play an important role in the adhesion and migration of leukocytes [45]. These mechanisms might all contribute to the stabilization of the carotid atherosclerotic plaques by attenuating the inflammation in patients treated with statins.

### Statin and infarct volume in stroke patients

Among patients presenting with strokes whose infarct volume was ≥ 1.0 cm^3^, those treated with statins had significantly smaller infarcts, compared with untreated patients (Fig. 7b). Since infarct volume was positively correlated with lumen thrombus in patients who had suffered a stroke, small thrombi in the statin-treated patients might have contributed to small infarct volumes. The smaller infarct volumes observed in patients treated with statins are also explained by earlier studies. One of the putative pleiotropic effects of statins is their effect on endothelial NO synthase, which, when absent in mice, is associated with larger infarcts size [46]. The effects of statins are likely mediated by Rho/Rho kinase since Rho kinase inhibitors upregulate endothelial NO synthase and increase the cerebral blood flow in mice [47].

Smaller infarct volumes in statin-treated patients might be associated with more favorable clinical outcomes. In other studies, the prescription of statin before the onset of strokes increased the survival at 7, 90 days and 1 year [48], as well as improved the functional outcome at 90 days [49].

### Non-significant pathological indices in comparative analysis

Statistically non-significant changes in plaque characteristics such as lipid core, fibrous tissue, calcifications and overall instability might be explained by too short an exposure to statins for these treatment effects to develop, as suggested by a previous experimental study [50]. Insignificant differences regarding foamy macrophages might be due to the nearly 50 days, on average, between onset of stroke and CEA, or to our choice of an imperfect methodology.

### Limitations of our study

The sample size of this retrospective, observational study, conducted at three medical centers, was small. Its results need to be confirmed by a study including a larger number of patients. Second, the type, dose and duration of statin administration was variable. Third, the patients included in our study underwent CEA after a relatively long median time interval after the stroke, while current professional practice guidelines recommend that patients presenting with symptomatic carotid stenoses undergo CEA within 14 days of the latest event. Therefore, further histopathological analyses may reveal a significantly less prominent infiltration by foamy macrophages in patients previously treated with statins. Fourth, we did not measure other biomarkers such as high-sensitivity C-reactive protein, which is associated with the risk of ischemic stroke and vascular events [51].

## Conclusion

This clinico-pathological analysis suggests that statin therapy stabilizes the carotid atherosclerotic plaques, which, in symptomatic patients undergoing CEA, may decrease the volume of infarcts. This stabilization of the plaques might be a pleiotropic effect, besides the lowering of lipids conferred by statins.

## Electronic supplementary material

Below is the link to the electronic supplementary material.

Supplementary material 1 (TIFF 337 kb)

Supplementary material 2 (TIFF 344 kb)
