# Supplementary figures and images for: Stabilization of symptomatic carotid atherosclerotic plaques by statins: a clinico-pathological analysis
Source: Heart Vessels. 2018 May 22;33(11):1311–24. doi: 10.1007/s00380-018-1193-6 (PMC6208692; doi:10.1007/s00380-018-1193-6)

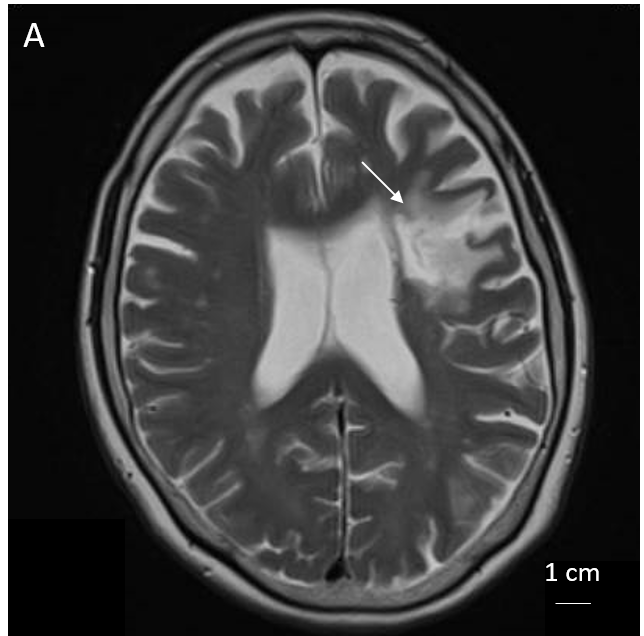

Supplement: Supplementary file 1 — Supplementary material 1 (TIFF 337 kb) [file 380_2018_1193_MOESM1_ESM.tif]

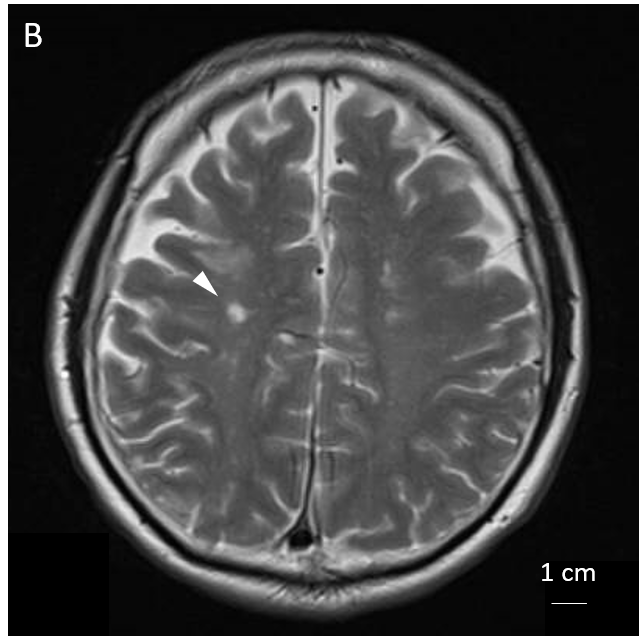

Supplement: Supplementary file 2 — Supplementary material 2 (TIFF 344 kb) [file 380_2018_1193_MOESM2_ESM.tif]
